# Apolipoproteins A-I, B, and C-III and Obesity in Young Adult Cherokee

**DOI:** 10.1155/2017/8236325

**Published:** 2017-04-03

**Authors:** Wenyu Wang, Piers Blackett, Sohail Khan, Elisa Lee

**Affiliations:** ^1^Center for American Indian Health Research, College of Public Health, University of Oklahoma Health Sciences Center, Oklahoma City, OK 73190, USA; ^2^Section of Diabetes and Endocrinology, Department of Pediatrics, Harold Hamm Diabetes Center, University of Oklahoma Health Sciences Center, Oklahoma City, OK 73104, USA; ^3^The Cherokee Nation, P.O. Box 948, Tahlequah, OK 74465, USA

## Abstract

Since young adult Cherokee are at increased risk for both diabetes and cardiovascular disease, we assessed association of apolipoproteins (A-I, B, and C-III in non-HDL and HDL) with obesity and related risk factors. Obese participants (BMI ≥ 30) aged 20–40 years (*n* = 476) were studied. Metabolically healthy obese (MHO) individuals were defined as not having any of four components of the ATP-III metabolic syndrome after exclusion of waist circumference, and obese participants not being MHO were defined as metabolically abnormal obese (MAO). Associations were evaluated by correlation and regression modeling. Obesity measures, blood pressure, insulin resistance, lipids, and apolipoproteins were significantly different between groups except for total cholesterol, LDL-C, and HDL-apoC-III. Apolipoproteins were not correlated with obesity measures with the exception of apoA-I with waist and the waist : height ratio. In a logistic regression model apoA-I and the apoB : apoA-I ratio were significantly selected for identifying those being MHO, and the result (*C*-statistic = 0.902) indicated that apoA-I and the apoB : apoA-I ratio can be used to identify a subgroup of obese individuals with a significantly less atherogenic lipid and apolipoprotein profile, particularly in obese Cherokee men in whom MHO is more likely.

## 1. Introduction

Since obesity predicts atherosclerotic cardiovascular disease (ACVD), it has significant worldwide health and economic implications [1]. This is particularly true in the Cherokee and other American Indian populations [2] in whom obesity is associated with the metabolic syndrome, which often precedes type 2 diabetes (T2D) [3]. Consequently it has become important to study association of obesity with apolipoproteins, since obesity-associated changes in lipid transport precede and predict subsequent insulin resistance and ultimately the development of ACVD and T2D [4, 5]. Therefore, we selected apolipoproteins known to predict atherosclerosis for study. We also proposed that the obese participants could be classified as two distinct groups based on the presence of metabolic complications including dyslipidemia [6] and that apolipoprotein levels might serve to identify differences between the metabolically healthy obese (MHO) and metabolically abnormal obese (MAO) groups.

Apolipoprotein B (apoB) represents the total number of apoB-containing lipoproteins [7] and is considered to be superior to LDL-C and non-HDL-C in predicting cardiovascular disease [8], whereas apolipoprotein A-I (apoA-I) has a known inverse association and low levels are associated with increased body mass index (BMI) [9]. Furthermore, the ratio of apoB to apoA-I (B : A-I ratio), representing the combination of two atherogenic processes, is an even stronger predictor [10]. Apolipoprotein C-III (apoC-III) is secreted with VLDL and becomes distributed among circulating lipoproteins [11] conferring harmful properties resulting in ACVD [3, 12]. LDL particles containing apoC-III are more atherogenic than particles without apoC-III [13] and apoC-III on non-HDL lipoprotein particles independently predicted recurrent coronary events [14] and progression of carotid intima-media thickness during treatment [15]. Following hepatic secretion of apoC-III as VLDL, its subsequent distribution on HDL particles may also be harmful, since HDL-apoC-III predicted angiographic progression of atherosclerosis in bypass grafts [16] and more recently HDL-apoC-III has been identified as a proatherogenic HDL subtype with loss of its anti-inflammatory properties [16].

Genetic deficiency [17] and targeted gene disruption [18] of apoC-III have been shown to be associated with protection from atherosclerosis [17]. However, the relative role of apoC-III's distribution on lipoproteins remains uncertain [19], and preliminary evidence suggests that obesity may play a central role in determining apoC-III levels, lipoprotein distribution, and clinical outcomes [20]. Consequently this study and analysis were done to examine association of obesity with apoB, apoA-I, and apoC-III content of both non-HDL and HDL.

## 2. Methods

With collaboration of the Cherokee Nation of Oklahoma, adults aged 20–40 years in the Cherokee Diabetes Study cohort residing in a 5-county area in northeastern Oklahoma participated in the study (*n* = 1051). Of this group 477 (45%) were obese, defined as having a BMI greater than or equal to 30. Nondiabetic participants were excluded according to American Diabetes Association criteria for fasting plasma glucose (FPG) defined as being greater than or equal to 126 mg/dl or being on medications for diabetes. Informed consent was obtained from each subject or his/her legal guardian, following approval of the Institutional Review Boards of the University of Oklahoma Health Sciences Center and the Cherokee Nation.

After obtaining clinical measurements, fasting blood specimens were collected for determining FPG, insulin, lipids, and apolipoproteins.

### 2.1. Lipids and Apolipoproteins

An Abbott VP-Super System automatic analyzer and commercial reagents were used to determine levels of glucose, cholesterol (Boehringer, Mannheim, Germany), and triglyceride (Miles Inc., Tarrytown, NJ) by enzymatic methodology. HDL-C was measured using the heparin-manganese precipitation procedure of the Lipid Research Clinics program and LDL-C was calculated by the Friedewald formula. ApoA-I, apoB, and apoC-III were determined by previously validated electroimmunoassays [21–23]. The apoC-III concentrations in whole plasma and heparin-manganese supernatant were determined by separate assays. ApoC-III in the precipitate was calculated by subtracting the supernatant value from the total plasma apoC-III.

### 2.2. Glucose and Insulin

Fasting insulin levels were determined in a National Institutes of Health core laboratory at the Endocrinology Department at the University of Chicago. Insulin was measured in serum samples using a competitive double antibody radioimmunoassay and glucose by an automated method using glucose oxidase (Alfa Wassermann, Inc., West Caldwell, NJ). The homeostasis index (HOMA-IR) was computed from the product of insulin (lU/mL) and glucose (mmol/L) divided by 22.5.

### 2.3. Blood Pressure, Waist Circumference, and Height Measurements

Three consecutive measurements of systolic blood pressure (SBP) and diastolic blood pressure (DBP) were performed on the right arm using a Baum mercury sphygmomanometer (W.A. Baum Co., Copiague, NY), and the average of the second and third measurements was recorded. The average of duplicate measures of the waist circumference and height was obtained using a nonstretchable linen tape for the waist circumference and a wall-mounted calibrated stadiometer for the height measurements.

### 2.4. Statistical Analysis

Means and standard deviations were estimated on all measurements on obese participants aged 20–40 years. Obesity was defined as having a BMI ≥ 30. Participants were separated by subgroups of metabolically healthy obese (MHO) individuals, defined as having none of four National Cholesterol Education Program (NCEP) metabolic syndrome components after excluding waist circumference. Metabolically abnormal obese (MAO) individuals had at least one of the four criteria. The mean difference of a variable between the two subgroups was calculated after adjusting for age and gender. Logarithmic transformation was used if a variable was not symmetrically distributed such as triglyceride. Spearman partial correlation was done between variables after adjusting for age and gender. To explore odds that an obese participant is classified as being MHO and distinguish from being MAO, logistic regression was used to select significant determinants with adjustment for age and gender. Statistical analyses were conducted with SAS (version 9.4), and a *P* < 0.05 was considered as significant.

## 3. Results

Measures of obesity (BMI, waist circumference, and waist to height ratio), blood pressure (systolic and diastolic), glucose homeostasis (fasting glucose, insulin, and the homeostasis index), lipids, and apolipoproteins were different between individuals being MHO and MAO with the exception of TC, LDL-C, and HDL-apoC-III (Table 1).

Significant correlations were observed between waist and waist to height ratio with apoA-I; diastolic blood pressure with HDL-apoC-III; and insulin and HOMA-IR with apoB, apoA-I, apoB : apoA-I ratio, and non-HDL-apoC-III (Table 2). Lipids showed expected correlations with apolipoproteins with exception of triglyceride with apoA-I.

In the logistic regression model for odds or probability of an obese individual being MHO, apoA-I and apoB : apoA-I ratio were significantly selected among apoB, apoA-I, apoB : apoA-I, LpA-I, LpA-I : A-II, non-HDL-apoC-III, and HDL-apoC-III into the model for identifying those with MHO among all the obese participants (Table 3). ApoA-I has a positive association while apoB : apoA-I ratio has a strong negative association with the odds of an obese individual being MHO. Assuming that the other variables in the model are the same, males had a more than four times chance of being MHO than females; and for one standard deviation higher apoA-I or apoB : apoA-I, the chance of being MHO is increased by 2.57 times or reduced by 69%, respectively (Table 3).

**Figure 1:**
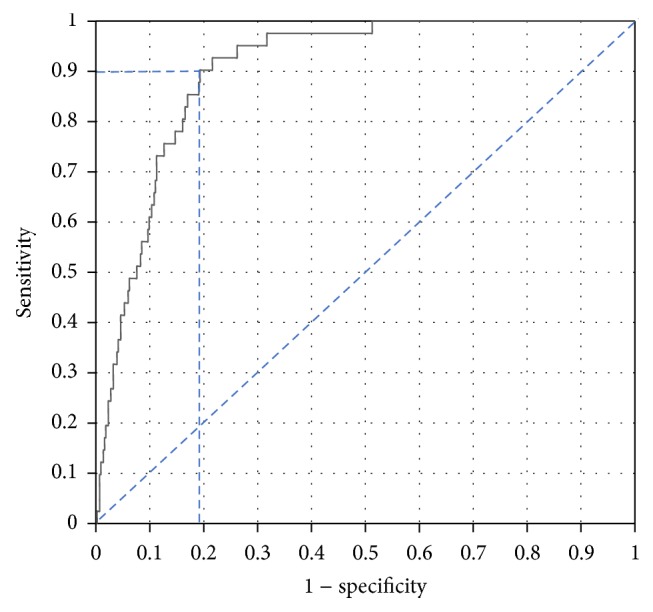
The receiver operating characteristic (ROC) curve from logistic regression selecting apoA-I and apoB to apoA-I ratio as identifying those with MHO among all obese individuals. The area under the ROC was 0.902 (that is, *C*-statistic = 0.902) indicating high discrimination of the model.

Figure 1 shows the receiver operating characteristics (ROC) curve derived from the logistic regression. The area under the curve (*C*-statistic) was 0.902, implying high discrimination ability of the model and consequently of both apoA-I and the apoB : apoA-I ratio in identifying those being MHO among all the obese participants. For a sensitivity of 90% the respective highest specificity is 81% and the corresponding cutoff probability is 0.0855 from the ROC curve (Figure 1).

## 4. Discussion

Paradoxically low non-HDL-apoC-III with relatively less atherogenic lipids and lipoproteins, resembling “metabolically healthy” obesity [6] with reduced cardiovascular risk [24] was present in a young adult Cherokee population. This group had reduced adiposity and less insulin resistance as has been observed in cross-sectional studies [25, 26]. This distinct entity may represent a transient phase in a sequence of worsening insulin resistance, a concept supported by the Atherosclerosis Risk in Communities (ARIC) study showing that risk factors increase after three years of follow-up when compared to a nonobese group [27]. Therefore, prescription of weight management for all obese patients, including the “metabolically healthy,” would be prudent to avoid atherogenic risk factor progression.

Insulin resistance, measured as the homeostasis index (HOMA-IR) and the fasting insulin level, correlated with apoB, with the apoB : apoA-I ratio, and inversely with apoA-I supporting known association of insulin resistance with lipoprotein transport [12]. Correlations of HOMA-IR and fasting insulin with non-HDL-apoC-III but not with HDL-apoC-III can be accounted for by an effect of insulin resistance on VLDL prior to apoC-III's transfer to HDL during lipolysis [28]. Since apoC-III has a known role in cardiovascular disease [14, 20] it may be a marker for atherosclerosis associated with obesity. Non-HDL-apoC-III is an independent predictor of atherosclerosis [15], and LDL containing both apoB and apoC-III is more atherogenic than LDL containing apoB alone [13], supporting an obesity-associated increase in risk attributable to apoC-III. Its atherogenic properties are further supported by adverse effects on the arterial wall including enhanced LDL binding to biglycan [29] and proinflammatory effects via nuclear factor kB-mediated VCAM-1 expression and monocyte adhesion [30]. We have previously shown that non-HDL-apoC-III is proportionate to the number of metabolic syndrome criteria [3] supporting contribution to cardiovascular risk in obesity, a proposed contributor to the syndrome [4]. Furthermore, hepatic insulin resistance influences apoC-III transcription [31, 32] beginning in childhood [32]. Also the apoC-III promotor contains a carbohydrate response element suggesting that dietary glucose and possibly saturated fatty acids increase transcription [33, 34] and contribute to increasing levels [35].

We confirmed known association of obesity with triglyceride and inverse association with HDL-C [36]. We observed an inverse correlation of the waist circumference and waist to height ratio, but not BMI, with apoA-I. Although height may influence the association, since it associates negatively with liver fat content but not with visceral fat mass [37], our observation supports the role of visceral fat in HDL metabolism in obesity and the concept that low HDL-C in obesity may be associated with apoA-I degradation and impaired cholesterol efflux from lipoprotein and cellular sources [38]. However, there is considerable heterogeneity in prediabetes phenotypes associated with obesity, and we did not evaluate non-alcoholic fatty liver disease (NAFLD) manifesting as increased hepatic fat. Furthermore, the presence of NAFLD could account for increased carotid intima-media thickness (cIMT) and a more dysregulated lipid and apolipoprotein profile [39]. Also male participants were much more likely to have MHO; men had a more than four times chance of being MHO than women. This finding is unique in obese Cherokee participants and different to sex differences for MHO among other populations that have favored females [40]. This observation can possibly be accounted for by differences in daily exercise or unknown factors [41]; however we have previously observed higher rates for the metabolic syndrome in 20–40-year-old males (30.9%) than in females (22.0%) consistent with higher overall risk for males [3].

Our findings emphasize the atherogenic role of low and dysfunctional HDL-C in obesity since low HDL-C is associated with risk for cardiovascular disease [42] even when LDL-C is lowered [43]. Both apoA-I and HDL-C are known to be low in obesity and the low levels are attributed to HDL's interaction with VLDL mediated by hepatic triglyceride lipase [44]. These metabolic events are in part attributed to insulin resistance [44] and account for our finding that MHO participants have less insulin resistance and higher apoA-I, possibly accounting for a less atherogenic effect, particularly when combined with lower apoB and the apoB : apoA-I ratio [45]. The high *C*-statistic and retention in the model support prediction of MHO and lower risk attributable to the apoB : apoA-I ratio [45, 46] and support use of the ratio as a measure of cardiovascular risk attributable to obesity.

## 5. Conclusions

Measurements of apoA-I, apoB, and apoC-III contained in HDL and non-HDL contribute information on risk and provide rationale for cholesterol, triglyceride, and apoC-III lowering strategies in obese individuals. Association of apoC-III in non-HDL, a known correlate of triglyceride, with insulin resistance and lipids supports early risk reduction treatments including lifestyle and lipid-lowering medications. Obese cases may present with relatively normal metabolic syndrome criteria and should be managed with caution since obese individuals may increase their risk for both atherosclerosis and T2D. ApoA-I was positively and apoB : apoA-I was negatively associated with the chance of being MHO, and both were good identifiers for being MHO suggesting that these apolipoprotein measures can be helpful in assessing risk in obese individuals, especially in men who have a much higher chance of being MHO than women.

## Figures and Tables

**Table 1:** Mean values, standard deviations, and differences (*P* < 0.05, bold type) between metabolically healthy obese (MHO) individuals, defined as those who are obese without any of four abnormal National Cholesterol Education Program (NCEP) metabolic syndrome components after excluding waist circumference, and metabolically abnormal obese (MAO) individuals defined as having one or more of the four abnormal criteria. Obesity was defined as body mass index (BMI) ≥ 30 for young adult Cherokee aged 20–40.

	MHO (*n* = 41)		MAO (*n* = 435)		*P*
	Mean	STD	Mean	STD	
*Obesity*					
Male	56.1%		41.1%		0.0643
BMI	33.87	3.87	36.81	6.90	**0.0096**
Waist (cms)	102.95	10.78	109.43	13.54	**0.0006**
Waist : height	0.62	0.05	0.66	0.09	**0.0022**
*Blood pressure* (mm Hg)					
SBP	118.59	7.57	122.88	13.01	**0.0009**
DBP	75.55	6.9676	79.41	9.6968	**0.0007**
*Insulin resistance*					
FPG (mmol/L)	4.55	0.55	4.85	0.62	**0.0047**
Insulin (*µ*U/ml)	114.60	62.45	181.24	116.0	**0.0002**
log⁡(Insulin)	4.59	0.57	5.03	0.57	**<0.0001**
HOMA-IR	3.88	2.23	6.58	4.51	**0.0001**
*Lipids* (mmol/L)					
TC	4.37	0.87	4.42	0.89	0.8534
TG	0.87	0.36	1.42	0.80	**<0.0001**
log⁡(TG)	4.25	0.4570	4.69	0.5366	**<0.0001**
LDL-C	2.65	0.81	2.83	0.78	0.2249
HDL-C	1.31	0.19	0.95	0.22	**<0.0001**
Non-HDL-C	3.05	0.88	3.47	0.88	**0.0040**
*Apolipoproteins* (*µ*mol/L)					
ApoB	1.56	0.37	1.75	0.40	**0.0023**
ApoA-I	2.56	0.31	2.15	0.32	**<0.0001**
ApoB : apoA-I	0.62	0.1542	0.83	0.2046	**<0.0001**
LpA-1	0.57	0.08	0.47	0.09	**<0.0001**
LpA-1 : A-II	1.99	0.24	1.68	0.24	**<0.0001**
Non-HDL-apoC-III	0.06	0.03	0.09	0.05	**0.0001**
HDL-apoC-III	0.09	0.02	0.09	0.02	0.4069

*P*, *P* value for difference between the two groups after adjusting for age and gender, and significant values (*P* < 0.05) are in bold font; STD, standard deviation.

**Table 2:** Spearman partial correlation between selected variables after adjusting for age and gender for obese young adult Cherokee aged 20–40 (*N* = 476).

	ApoB		ApoA-I		ApoB : apoA-I		Non-HDL-apoC-III		HDL-apoC-III	
	*R*	*P*	*R*	*P*	*R*	*P*	*R*	*P*	*R*	*P*
*Obesity*										
BMI	−0.012	0.803	−0.085	0.074	0.047	0.326	0.052	0.275	−0.058	0.217
Waist	−0.029	0.542	−0.123	**0.009**	0.044	0.349	0.070	0.140	−0.062	0.189
Waist : height	−0.015	0.755	−0.109	**0.021**	0.056	0.241	0.049	0.304	−0.077	0.106
*Blood pressure*										
SBP	0.037	0.429	0.062	0.192	−0.008	0.863	−0.003	0.956	0.038	0.423
DBP	0.049	0.296	0.011	0.824	0.035	0.464	0.038	0.428	0.099	**0.036**
*Insulin resistance*										
FPG	0.068	0.154	−0.037	0.441	0.066	0.164	0.027	0.568	0.024	0.610
HOMA-IR	0.112	**0.018**	−0.106	**0.025**	0.173	**0.000**	0.183	**0.000**	0.037	0.441
Insulin	0.107	**0.023**	−0.102	**0.030**	0.172	**0.000**	0.190	**0.000**	0.035	0.463
*Lipids*										
TC	0.814	**<0.001**	0.340	**<0.001**	0.505	**<0.001**	0.510	**<0.001**	0.369	**<0.001**
LDL-C	0.725	**<0.001**	0.178	**0.000**	0.528	**<0.001**	0.299	**<0.001**	0.163	**0.001**
HDL-C	−0.143	**0.002**	0.759	**<0.001**	−0.562	**<0.001**	−0.299	**<0.001**	0.192	**<0.001**
Non-HDL-C	0.862	**<0.001**	0.132	**0.005**	0.672	**<0.001**	0.588	**<0.001**	0.316	**<0.001**
TG	0.602	**<0.001**	−0.090	0.057	0.574	**<0.001**	0.886	**<0.001**	0.449	**<0.001**

*P*, *P* value for Spearman partial correlation after adjusting for age and gender, and significant values (*P* < 0.05) are in bold font; *R*, Spearman partial correlation.

**Table 3:** Logistic regression model for odds or probability of an obese participant being metabolically healthy obese (MHO).

Variable	Estimate	SE	*P*	Unit^*∗*^	OR	95% CI
Intercept	−4.245	1.888	0.0246			
Age	−0.049	0.036	0.1770	5	0.78	0.54, 1.11
Male versus female	1.433	0.401	**0.0004**		4.19	1.94, 9.46
ApoA-I	0.055	0.012	**<0.0001**	17.19	2.57	1.75, 3.90
ApoB : apoA-I	−5.594	1.361	**<0.0001**	0.21	0.31	0.17, 0.53
*C-statistic*	0.902					

^*∗*^One standard deviation used for apoA-I and apoB : apoA-I.

CI, confidence interval; *C*-statistic, the area under the receiver operating characteristic curve; Estimate, estimated coefficient; OR, odds ratio; *P*, *P* value; SE, standard error.

## Abbreviations

Apo: Apolipoprotein before A-I, B, and C-III
BMI: Body mass index
FPG: Fasting blood glucose
TC: Total cholesterol
TG: Triglyceride
LDL-C: LDL-cholesterol
HDL-C: HDL-cholesterol
apoB : apoA-I ratio: Apolipoprotein B to A-I ratio
LpA-1 : A-II: Lipoproteins containing apoA-I and apoA-II
SBP: Systolic blood pressure
DBP: Diastolic blood pressure
Log: Logarithm
HOMA-IR: Homeostasis index for insulin resistance
WHR: Waist to height ratio
Waist: Waist circumference
Non-HDL-apoC-III: ApoC-III in non-HDL
HDL-apoC-III: ApoC-III in HDL.
